# Effectiveness of guided self-help in decreasing expressed emotion in family caregivers of people diagnosed with depression in Thailand: a randomised controlled trial

**DOI:** 10.1186/s12888-015-0654-z

**Published:** 2015-10-21

**Authors:** Terence V. McCann, Wallapa Songprakun, John Stephenson

**Affiliations:** 1 Centre for Chronic Disease, College of Health and Biomedicine (Discipline of Nursing), Victoria University, PO Box 14428, Melbourne, VIC 8001 Australia; 2 McCormick Faculty of Nursing, Payap University, Chiang Mai, Thailand; 3 School of Human and Health Sciences, University of Huddersfield, Huddersfield, West Yorkshire UK

**Keywords:** Cognitive behaviour therapy, Depression, Expressed emotion, Guided self-help, Primary caregivers, Self-help manual

## Abstract

**Background:**

High expressed emotion (EE) can extend the duration of illness and precipitate relapse; however, little evidence-based information is available to assist family caregivers of individuals with depression. In the present exploratory study, we examined the effectiveness of a cognitive behaviour therapy (CBT) based guided self-help (GSH) manual in decreasing EE in caregivers of people with depression, in Thailand.

**Method:**

A parallel group randomised controlled trial was conducted, following CONSORT guidelines, with 54 caregivers who were allocated equally to GSH or control group (standard outpatient department support). In addition, both groups were contacted weekly by telephone. EE was assessed, using the Family Questionnaire (FQ), at baseline, post-test (Week 8) and follow-up (Week 12).

**Results:**

FQ scores at baseline indicated that both groups had similar, though moderately high level of EE. However, between baseline and post-test EE scores decreased markedly in the intervention group, but in contrast, they increased slightly in the control group. Between post-test and follow-up, little change took place in the EE scores of either group. Overall, the intervention group recipients of GSH showed a significant decrease in EE whereas the control group recipients of standard outpatient department support reported a slight increase in EE.

**Conclusion:**

These findings provide preliminary evidence that GSH is beneficial in reducing EE in caregivers, which is advantageous to family members with depression and caregivers. The approach may be used as an adjunct to the limited outpatient department support given to caregivers by mental health professionals and, perhaps, to caregivers who do not attend these departments.

**Trial registration:**

Australian and New Zealand Clinical Trials Registry https://www.anzctr.org.au/Trial/Registration/TrialReview.aspx?id=366639. Registered 21 July 2014.

## Background

The prevalence of depression is increasing rapidly in Thailand, where it is predicted to become the dominant mental health problem in the country [1]. To illustrate, between 1997 and 2007 there was over a six-fold increase in its prevalence (from 56 to 346 per 100,000 population) [1, 2]. In addition to affecting the person directly, depression has a significant adverse effect on other family members, especially primary caregivers' physical [3], emotional [4–6], social and financial [7] well-being. This, in turn, can contribute to high EE, with caregivers making critical comments to, and being hostile, emotionally over-involved and over-protective toward, the family member with depression. High EE in caregivers is predictive of longer duration of illness and increased relapse rates in mental illnesses such as depression [8–10] and schizophrenia [11]. In particular, people with depression living in high EE families are susceptible to relapse even when they are recipients of relatively few critical comments [8, 10, 12]. A considerable amount of face-to-face contact (exceeding 35 h per week) with a family member with high EE increases the risk of relapse; however, frequent contact with a family member with low EE (conservative criticism, emotional warmth, supportive comments) seems to be protective [13]. Another consideration is that the effect of EE on illness duration and relapse can vary from culture-to-culture [13]. This was evidenced in a US study of 42 patient/family member dyads with schizophrenia, where patients of white and Latino family members with high EE were perceived as more critical, whereas no significant association was found in patients of black family members with high EE [14]. Therefore, factors such as cultural and ethnic values [14], kinship and family attitudes toward mental illness and broader cultural influences [13] may influence how people with mental illness perceive high EE from relatives.

Family caregiver interventions can reduce EE and strengthen caregivers' self-esteem and coping, and improve outcomes for the family member with mental illness [11]. CBT incorporated within a GSH manual may be one way of decreasing EE in a family caregiver supporting a member with depression. GSH is a form of therapy-based self-help, where reading and written information are included within a workbook [15, 16]. Within the context of caregivers of family members with depression, providing a GSH manual for individuals with this illness may also be beneficial to their caregivers’ EE. The manual provides a framework to help caregivers understand their family member’s illness, to be more empathetic, and to have lower EE. In so doing, it equips caregivers to cope better, improves communication with and supports people with depression to deal with their illness [17]. Another consideration about GSH is it can be used without the involvement of mental health professionals [16, 18]; however, it seems to work better if used in association with other therapeutic approaches [15], such as occasional contact with mental health professionals.

A systemic review and meta-analysis [18] has compared the efficacy of GSH with regular face-to-face psychotherapy for depressive and anxiety disorders. The findings showed that the outcomes of these approaches were similar, and there was no difference in attrition rates of both methods. While a significant number of self-help studies have been carried out, several have concentrated on people with depression [17], but few have focused primarily on family caregivers [19, 20], and specifically in reducing EE in caregivers of people with depression. Moreover, no randomised controlled trials (RCTs) have been undertaken to evaluate the efficacy of GSH in reducing EE in primary caregivers of individuals with depression, particularly in a developing country such as Thailand, which is experiencing a marked increase in the prevalence of depression [1, 2]. An evaluation of GSH is also opportune because that country's mental health services are provided predominantly in psychiatric hospitals in large cities, with limited access to services in rural areas, and minimal specialised support for family caregivers [21]. In addition, because the effect of EE can vary between cultures [13], it is important to examine this construct within a Thai cultural context.

In the current exploratory study, we aimed to evaluate if primary caregivers of adults receiving outpatient department treatment for depression, who took part in a GSH program, had a lower level of EE than a wait-list control group recipients of standard support for caregivers. A primary caregiver is defined as the 'main person (aside from health, social, or voluntary care provider) responsible for assisting with activities of daily living, supporting and advocating on behalf of' ([3], p.382) an individual with depression.

## Method

### Design

This parallel-group design followed the CONSORT guidelines [22] and checklist [23] for conducting RCTs. A computerised random number generator was used to allocate caregivers randomly, using block randomisation, 1:1 to intervention and wait-list control group. Allocation was concealed: it was done off-site, in Australia, and was undertaken by a researcher who was not directly involved in recruitment. Randomisation was then emailed to the researcher who allocated participants to intervention and control groups. After trial commencement, no change of original protocol occurred.

### Participants and recruitment

Primary caregivers of patients attending a psychiatric outpatient department for treatment of depression (who had consented to take part in a separate RCT of GSH [24, 25]) participated. Caregivers were recruited through mental health clinicians at Suan Prung Psychiatric Hospital outpatient department in Chiang Mai City in northern Thailand. Clinicians provided brief to them information about the study; those who expressed interest in participating were referred to the researcher, who provided detailed information about participation.

#### Inclusion criteria

(a) primary caregiver of an adult receiving outpatient department treatment for moderate depression ((F32.1), ICD-10 classification) [26], (b) aged 18–60 years, able to read and write in Thai, and (c) had a telephone at home.

#### Exclusion criterion

A caregiver currently receiving treatment for an acute episode of mental illness.

### Sample size and power

IBM® SPSS® SamplePower (Vers. 2.0) was used to calculate sample size, which showed that a standardised difference between both groups of 0.8 (considered an effect size of clinical significance) could be achieved with 80 % power with a sample of 52, with a type I error probability of 0.05. To allow for some attrition, the sample size was increased to 56.

### Measure

The Family Questionnaire (FQ) [27], a 20-item self-report instrument assessing EE directed by, in this instance, a primary caregiver towards a family member with depression, was used. It comprises two sub-scales (critical comments, emotional over-involvement) rated on a 4-point Likert scale, ranging from 1 (never/very rarely) to 4 (very often). Critical comments are adverse statements about the behaviour or personality of the person with depression, while emotional over-involvement comprises over-protectiveness, over-intrusiveness and over-identification with the person, by the caregiver [28]. Higher scores (maximum of 80) signify high levels of EE, which are associated with a greater likelihood of relapse; lower scores (minimum of 20) indicate low levels of EE (supportive comments and lack of over involvement), which are associated with a reduced risk of relapse [29]. The FQ has been validated against the Camberwell Family Interview, which is regarded as the benchmark or 'gold standard' measure of EE [27]. In the current study, Cronbach's alpha was used to assess internal reliability of the FQ at baseline, showing good reliability (α = 0.900).

As no pre-existing Thai version of the FQ existed, the WHO guidelines for translating instruments [30] was used to translate it into Thai. Research outcomes were assessed at baseline (Week 0), post-test (Week 8) and follow-up (Week 12).

### Procedure

The study was undertaken in caregiver participants' homes in several provinces in the upper northern part of Thailand, including Chiang Mai City. Control group participants received standard outpatient department support when they accompanied the family member with depression to the department for consultations and prescription of antidepressant, or a combination of antidepressant and anti-anxiety, medications. At the same time, the caregiver received limited information and support from mental health clinicians about how to support the family member with depression.

Intervention group participants were provided with a copy of The Good Mood Guide: A self-help manual for depression [31, 32], which had been translated into Thai. The manual was based on self-help and CBT principles and contained eight modules: (i) Summarises depression. Helps the person to assess depression and distress levels and promotes physical exercise. (ii) Emphasises the importance of maintaining social contact and physical activity. (iii) Assists carers to comprehend how people with depression feel and think and how they can recognise and label automatic thoughts and link situations and emotions to events in their lives. (iv) Shows how to change the person with depression’s thought patterns from negative to positive. (v) Highlights how healthy living, social support and problem-solving can help the person to change their behaviour and overcome their depression. (vi) Provides skills to improve the person with depression’s sleep pattern and how to maintain positive thoughts, emotions and behaviours. (vii) Shows carers how the person with depression can practise progressive muscle relaxation to deal with stress and enhance time management. (viii) Emphasises to carers how previously learned skills in changing behaviours, thought challenging and learning how to cope with difficult situations can assist the person with depression.

Caregivers were shown how to use the manual and requested to complete one module each week. On average, it took two hours to finish each module, which comprised reading and writing and some homework exercises. While the manual was developed initially as GSH for individuals with depression, caregivers could also use it.

Both groups were contacted by telephone each week, for approximately 5 min, by a researcher. The purpose of the call was to answer caregivers’ questions, provide limited support, and, for the GSH group only, to answer caregivers’ questions about using the manual.

### Treatment fidelity

Treatment fidelity was assessed during the weekly telephone calls by asking GSH group participants a prearranged set of questions about the content of the module completed that week.

### Ethical considerations

Victoria University, Melbourne and the Mental Health Department, Public Health Ministry of Thailand, Bangkok human research ethics committees gave approval to conduct the study. All participants gave written, informed consent.

### Data analysis

IBM® SPSS® Statistics Version 20.0 was used to analyse the data, by a researcher who was blind to group allocation. An insignificant amount (<0.5 %) of FQ item scores were recorded as missing. Descriptive statistics were used to analyse demographic data, with summary characteristics provided across the whole cohort and for both treatment groups. The balance of the groups at baseline was monitored for imbalances of any demographic characteristics across groups. Established procedures were followed whereby decisions on covariate adjustment in the case of baseline imbalance were made by prior knowledge of an influence on an outcome. A mixed analysis of variance (ANOVA) technique, incorporating between-participants and within-participants factors, was adopted to assess the effect of grouping, time and interaction effects. Effect sizes were evaluated using the partial eta-squared statistic. Intention-to-treat analyses were undertaken.

## Results

**Fig. 1:**
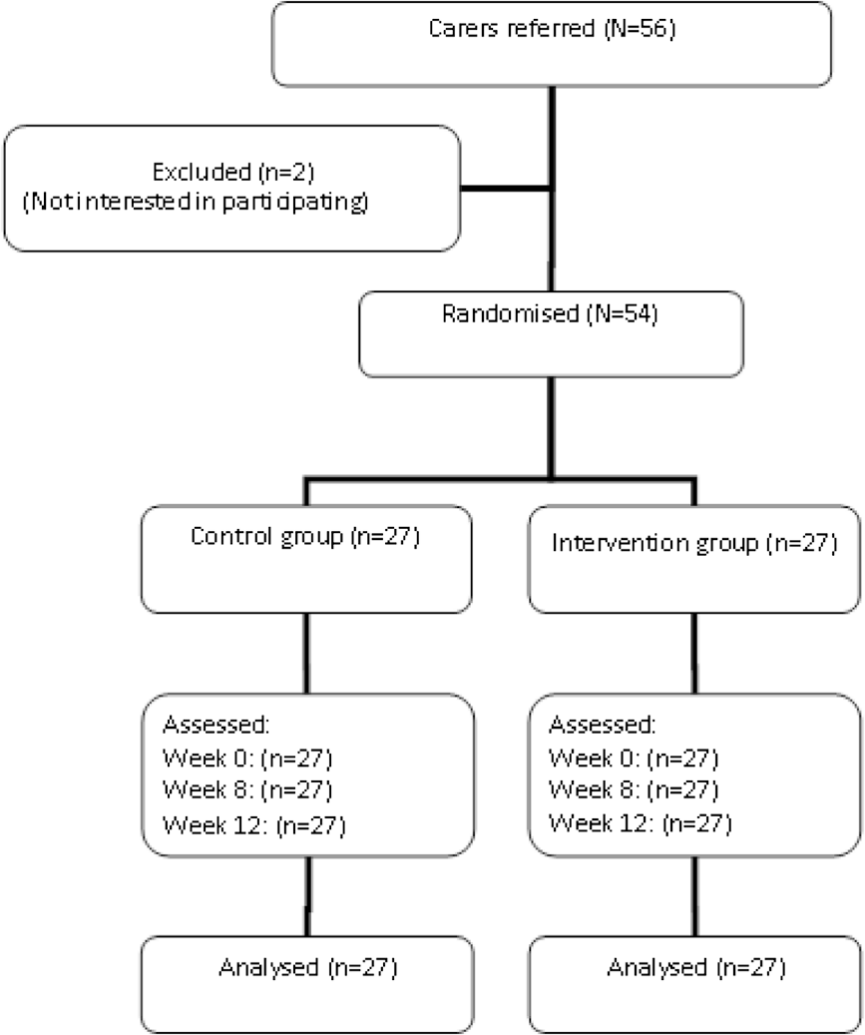
Flow of caregiver participants through each phase of the study

Fifty-six caregivers met the inclusion criteria for the study and, of these, two were excluded because they were unwilling to participate. Subsequently, 54 were randomised to an intervention (*n* = 27) or control (*n* = 27) group; none withdrew (Fig. 1).

Slightly more females than males participated, and the mean age of participants was 41 years (SD 8.95). Most participants were married or in a *de facto* relationship, and in paid employment. Slightly more were educated to primary or high school level, while the remainder were educated to further or higher education level. Analysis of group characteristics at baseline indicated good balance across groups with respect to the key covariates; indicating the effectiveness of the randomisation procedure. The moderate imbalance in a single variable (gender) was consistent with expectations from a study of this size measured on several factors and covariates at baseline. No prior knowledge linking gender to the outcome variable existed; in the case of other variables baseline imbalance was such that possible controlling was not necessary (Table 1).

**Table 1:** Participants' demographic information

Variable	Control	Intervention	Influence on outcome	All
	(*n* = 27)	(*n* = 27)		(*n* = 54)
Gender			No	
Male	11 (40.7 %)	15 (55.6 %)		26 (48.1 %)
Female	16 (59.3 %)	12 (44.4 %)		28 (51.9 %)
Marital status			N/A	
Single/divorced/widowed	6 (22.2 %)	8 (28.6 %)		14 (25.9 %)
Married/ *de facto*	21 (77.8 %)	19 (71.4 %)		40 (74.1 %)
Employment status			N/A	
Studying/retired/home duty	7 (25.9 %)	7 (25.9 %)		14 (25.9 %)
Employed	20 (74.1 %)	20 (74.1 %)		40 (74.1 %)
Highest educational level			N/A	
High school or below	15 (55.6 %)	13 (48.1 %)		28 (51.9 %)
Further or higher education	12 (44.4 %)	14 (51.9 %)		26 (48.1 %)
Age (years)	Mean (SD)	Mean (SD)	N/A	Mean (SD)
	41.0 (9.78)	41.0 (8.22)		41.0 (8.95)

Measurement of FQ scores at baseline indicated that both groups had similar, though moderately high levels of EE. However, between baseline and post-test EE scores decreased markedly in the intervention group, but in contrast, they increased slightly in the control group. Between post-test and follow-up, little change took place in the EE scores of either group (Table 2).

**Table 2:** Mean and standard deviation (SD) for FQ scores at each time point

Variable	Control (*n* = 27)	Intervention (*n* = 27)	All (*n* = 54)
	Mean (SD)	Mean (SD)	Mean (SD)
Baseline	48.9 (9.4)	48.6 (10.8)	49.1 (10.4)
Post-test	50.8 (6.7)	38.7 (6.5)	44.8 (8.9)
Follow-up	50.6 (7.3)	38.3 (6.3)	44.6 (9.2)

The repeated measures ANOVA showed evidence of a significant interaction between time and group in FQ scores (F_1.72,102_ = 11.3; *p* < 0.001); with a partial-η^2^ statistic of 0.181; indicating a large effect. This analysis incorporated the Greenhouse Geisser correction to the degrees of freedom, as Mauchly's test indicated evidence to reject the hypothesis of sphericity.

A simple main effects analysis (incorporating the Greenhouse Geisser correction factor) provided evidence for a significant difference in FQ scores between the measured time points (F_1.72,102_ = 5.45; *p* = 0.008); with a partial-η^2^ statistic of 0.097; indicating a medium effect. Pairwise comparisons between the intervention and control groups (incorporating the Sidak correction for multiple comparisons) indicated FQ scores to be significantly different between baseline and follow-up (*p* = 0.007; 95 % confidence interval for the difference (0.99, 7.53)); but not significantly different between baseline and post-test (*p* = 0.070; 95 % confidence interval for the difference (−0.24, 8.21)) and between post-test and follow-up (*p* = 0.995; 95 % confidence interval for the difference (−2.83, 3.38)). Hence, the larger time interval, from baseline to follow-up, is needed for the inference of a statistically significant difference between groups: neither of the intermediate steps, from baseline to post-test and post-test to follow-up, was associated with significant differences.

**Fig. 2:**
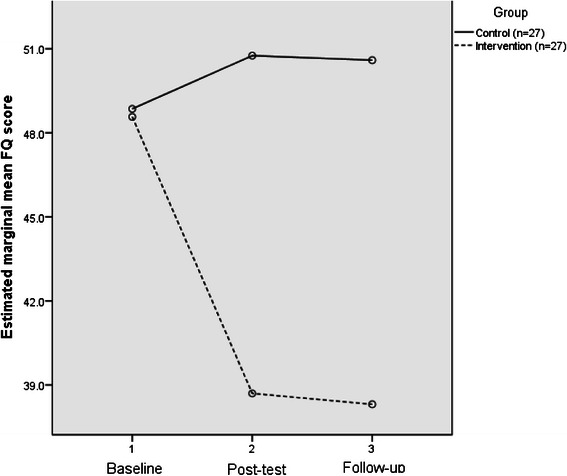
Estimated marginal mean FQ scores: control and intervention groups

The effect of group was also detected to be statistically different between groups (F_1,51_ = 32.9; *p* < 0.001; 95 % confidence interval for the difference (5.34, 11.1)). A partial-η^2^ statistic of 0.392 was computed for the group effect; revealing a large effect. The marginal mean FQ scores for the control and intervention groups were assessed at each of the three time points. It can be seen that FQ scores increased between baseline and follow-up in the control group, and reduced in the intervention group, indicative of a time-group interaction (Fig. 2).

### Treatment fidelity

All intervention group participants finished the GSH manual, while 2 (7.4 %) re-read sections after completing post-test data collection. Eight (29.6 %) completed the written requirement, 6 (22.2 %) completed around three-quarters, and 9 (33.3 %) finished half the written component. In addition, all the intervention group participants engaged in the weekly telephone call.

## Discussion

The main findings of this exploratory study, which evaluated the effectiveness of a GSH manual in decreasing EE in family caregivers of people with a diagnosis of moderate depression, in Thailand, are that both groups were experiencing moderately high levels of EE at baseline. However, intervention group recipients of GSH reported a significant decrease in EE, from baseline to post-treatment and baseline to follow-up but not between post-test and one-month follow-up. In contrast, the EE levels of the control group, who received standard outpatient department-provided information and support, increased between baseline and post-test and only decreased marginally between post-test and follow-up. Overall, the differences between the two groups may be attributable to the beneficial effects of GSH in reducing EE in the intervention group of caregivers; hence, the findings are of clinical significance. The findings are also consistent with those of systematic reviews and meta-analyses highlighting the effectiveness of GSH for depression [18, 33].

A family member's depressive symptoms can have detrimental effects on a caregiver's well-being [34, 35], which, in turn, can have adverse consequences for EE. High EE can increase the duration of the depressive episode and increase susceptibility to relapse [8, 10, 12]. It can also compromise caregiver coping and heighten stress within the family. An implication of this is it can extend the duration of the episode of depression and contribute to unfavourable outcomes for the family member with depression [36], and for the caregiver. Similarly, living in a household characterised by lack of support and high levels of stress heightens the possibility of poorer prospects for recovery and increases the likelihood of relapse in comparison to residing in a family with good support and low amounts of stress [13].

Thai family culture, which is characterised as mainly extended family-type, places an expectation on families to care for sick relatives, irrespective of whether they have a medical or mental illness [37]. This cultural imperative does not mean EE is unimportant; rather, it has implications for illness duration and relapse [13]. The finding of our study that both groups of participants were experiencing moderately high levels of EE at the beginning of the study is an important finding because individuals with depression who reside in high EE families are particularly prone to relapse, even when they receive relatively few critical comments [8, 10, 12]. The significant decrease in EE levels of the intervention group is associated with a reduced risk of relapse [29]. As such, this suggests that GSH assisted caregivers by strengthening their self-esteem and coping [11], providing information, facilitating insight, encouraging discussion, and offering a choice of feasible ways to address problems when supporting the family member with depression [17, 18].

This is the first exploratory RCT in Thailand to evaluate the effectiveness of GSH in reducing EE in caregivers. There are several advantages in using this approach. It can be used to augment the limited amount of information and support provided to caregivers in this situation by mental health professionals [15]. It is straightforward to use [31]; however, it requires some level of reading and writing ability. It is also a cost-effective and accessible method [38], especially in developing countries like Thailand, where mental health services are provided mainly in psychiatric hospitals in large cities, and where psychiatric rehabilitation is often under-funded and the needs of caregivers are accorded a low priority [39]. As many Thais live in rural areas and have to endure the cost and inconvenience of travelling long distances to obtain mental health services [21], GSH offers some support to caregivers in this situation, especially in light of the rapidly increasing prevalence of depression in the country [1, 2]. Moreover, support for family caregivers can reduce EE and enhance caregivers' coping and self-esteem, and lead to better outcomes for the affected family member [11], with depression. There is also scope for using this type of self-help approach to reduce EE in caregivers of family members with other forms of mental illness, such as psychotic [40], anxiety and eating disorders. Furthermore, the overall adherence rate in the present study compares favourably with that in the Songprakun and McCann [41] study of people with depression in Thailand.

### Limitations

There are several limitations to this exploratory study. First, the researcher who completed the FQ assessment with participants was not blinded to participant allocation to groups. In our opinion, while this did not affect the findings adversely, it is, regardless, a potential limitation. Furthermore, as the FQ is a self-report instrument more studies are needed using this questionnaire in Thailand, validated against, for example, the Camberwell Family Interview, the ‘gold standard’ measure of EE [42]. Second, the benefits of GSH may be limited where caregivers have minimal or no reading and/or writing ability, or the time, motivation and/or perseverance to read. Third, because the caregivers completed the manual in 8 weeks, this may explain why there was no significant reduction in EE between post-test (Week 8) and follow-up (Week 12). In addition, the 12-week follow-up period may have been too short to establish if the reduction in EE could be maintained in the longer term. Finally, the findings may also have been attributable to the relatively small sample of caregivers.

## Conclusion

The findings of our study provide initial evidence that GSH produces a significant reduction in EE in family caregivers of people with moderate depression when compared to wait-list control group recipients of information and support given by mental health professional at a psychiatric outpatient department. While GSH can be used to supplement standard support given by mental health professionals in outpatient departments, its real potential is it has good penetration and reach, especially in developing countries like Thailand where limited support is provided to family caregivers, especially those living outside major cities with little access to mental health services. Additional research is required to evaluate the effectiveness of this approach with a larger group of participants and with a longer intervention and follow-up period, and with caregivers who do not routinely access psychiatric outpatient departments. One approach to sustaining a reduction in EE is to provide intermittent booster sessions to caregivers. Furthermore, joint research is required, involving family caregivers and individuals diagnosed with depression, to evaluate if intervening early and adopting a shared approach with GSH not only reduces caregivers' EE but shortens the duration of illness and prevents relapse, in individuals with depression.

## Abbreviations

CBT: Cognitive behaviour therapy
EE: Expressed emotion
FQ: Family Questionnaire
GSH: Guided self-help
